# Trends of HIV/AIDS in Pregnant Women Presenting to a Tertiary Care Setting

**DOI:** 10.7759/cureus.73539

**Published:** 2024-11-12

**Authors:** Mishal Maqbool, Naushin Farooq, Iqra Shehzadi, Hafiza Faiza Mushtaq, Zain Maqbool, Maqbool Hussain, Ruhina Adil, Habib Ur Rehman Khalil

**Affiliations:** 1 Department of Medicine, Federal Government Polyclinic Hospital, Islamabad, PAK; 2 Department of Obstetrics and Gynaecology, Federal Government Polyclinic Hospital, Islamabad, PAK; 3 Department of Medicine, Fatima Memorial Hospital (FMH) College of Medicine and Dentistry, Lahore, PAK; 4 Medicine, Pakistan Institute of Medical Sciences, Islamabad, PAK; 5 Medicine, Rawal Institute of Health Sciences, Islamabad, PAK; 6 Paediatric Medicine, Pakistan Institute of Medical Sciences, Islamabad, PAK; 7 Medicine, Jinnah Sindh Medical University, Karachi, PAK; 8 Department of Gastroenterology, Lahore General Hospital, Lahore, PAK

**Keywords:** baby, hiv/aids, pregnancy, public health, vertical transmission

## Abstract

Introduction: According to WHO, there are around 35 million adults living with HIV/AIDs worldwide. Of these, around 1.5 million are pregnant women. There is a high risk of vertical transmission of HIV infection. Preventing mother-to-child transmission of HIV infection is one of the foremost challenges in public health. This study was done to assess the rate of vertical transmission of HIV infection in pregnant women presenting to a tertiary care setting.

Methods: A retrospective analysis of 15 years of hospital data from 2008 to 2022 was conducted in the Mother and Child Health Care (MCH) Center, Pakistan Institute of Health Sciences, Islamabad. The demographic, clinical, and delivery outcomes of the mother were retrieved from hospital records. Data was analyzed in SPSS software, version 22.0 (IBM Corp., Armonk, NY).

Results: A total of 197 pregnant women with HIV/AIDS presented in 15 years. The mean age was 29.5 ± 6.4 years, and the majority was between 21 and 30 years 114 (57.6%). When HIV RNA load was assessed in study women, it was witnessed that 67 (34.1%) had undetectable load while 130 (65.8%) had detectable viral load. A significant linear trend of increase in rates of women with HIV in pregnancy was noted from 2008 (3.0%) to 2022 (15.7%). Vertical transmission of HIV was found in two (1.0%) of study cases.

Conclusion: The linear trend of increase in HIV in pregnancy was noted. The rate of vertical transmission was found to be 1%. It proves the role of Highly Active Antiretroviral Therapy (HAART) therapy in reducing the risk of HIV transmission from mothers to babies.

## Introduction

Human Immunodeficiency Virus (HIV) infection has remained a major global public health concern. According to the World Health Organization's HIV prevalence report (2020), about 37.7 million people were reported HIV positive worldwide, and of these, around 1.3 million were pregnant women [1]. The American Foundation for AIDS reported a 51% incidence of HIV infection in women among all HIV-infected adults and poses a serious life threat worldwide [2]. The main route of HIV infection is an exchange of infected body fluids or unprotected intercourse with an infected person. Transmission of HIV from mother to child, also called vertical HIV transmission may occur through placental fluid transfer or later by breastfeeding [3].

The risk of mother-to-child transmission could be prevented by prenatal screening of infected pregnant women, early diagnosis, treatment with antiretroviral therapy (ART), prophylaxis of newborns with weight-dependent ART dose, and infant formula feeding [4]. HIV-infected pregnant women have higher chances of a shorter gestation period and low birth weight of infants which is highly associated with the incidence of HIV infection in infants [5].

The use of ART suppresses HIV viral load to undetectable levels and enhances the cluster of differentiation 4 (CD4) T-cell count, resulting in decreased morbidity and mortality among the infected patients [6,7]. Proper ART administration during pregnancy and prophylactic ART administration in infants significantly reduces the rate of vertical HIV transmission [8]. Currently, Pakistan has an estimated 183,705 people living with HIV. In Pakistan, the majority of HIV-infected cases are predominately among adults with high-risk behaviors, i.e., involved in drugs and unprotected sexual activities with transgender and sex workers. 

In 2018, the estimated number of HIV-infected population in Pakistan was 160,000 people, of which 2,2% were children below 15 years of age. At first, 14 children were reported HIV positive in April 2019, followed by 930 positive cases within the next three months [9,10]. Marcu et al. reported a 3.5% mother-to-child transmission rate of HIV. A statistically significant correlation between the level of maternal HIV viremia and perinatal HIV transmission (p = 0.01) was reported [11]. Since there is a lack of dissemination of overall HIV data, the case of pregnant women is altogether neglected. In Pakistan, due to social taboos, non-acceptance, and fear of discrimination and shaming of the affected people, most of the community hide this ailment and most of the cases do not opt for screening as well. This study aimed to determine the trends of frequency of HIV/AIDS in pregnant women and vertical transmission from mother to child.

## Materials and methods

This retrospective study was conducted at the Gynaecology and Obstetrics Department, Mother and Child Health Care (MCH) Center, Pakistan Institute of Medical Sciences (PIMS), Islamabad, after approval from the Ethics Review Board, PIMS, Islamabad (approval number F.3-2/2022 (ERRB)/PIMS). The medical record of patients comprising the last 15 years from January 2008 to December 2022 was reviewed and noted. Data collection was completed six months after approval of the synopsis. There were 197 mothers with HIV/AIDS who delivered in the past 15 years. The study inclusion criteria included all pregnant women whose tests were found positive for HIV during pregnancy. These women were scheduled to deliver their babies at MCH Center, PIMS Hospital Islamabad. The newborns born to HIV-positive mothers at the MCH Center, PIMS, Islamabad, were recorded. All those cases were excluded where the pregnancies did not result in live birth of children or had abortions. Incomplete medical records of HIV-positive pregnant women and those who reported leaving ART therapy were excluded from the study.

The demographic and clinical details of the patients and newborns were filled in a pre-designed proforma. The electronic record of the Gynaecology and Obstetric department of PIMS, Islamabad, was accessed to get the records of patients keeping the ethical terms under consideration. Consent forms are usually filled out by all the patients at the time of admission. The demographic details, such as the age and clinical data of the mothers were accessed. Undetectable HIV RNA Load was defined as less than 50 copies/ml in the sample, while detectable HIV RNA load was defined as measurable HIV RNA in the sample.

We followed the guidelines set by the National Institute of Health under the National AIDS Control Program [12]. Services offered included pre-ART services, ART services, diagnostic services, HIV confirmation, baseline investigations, CD4 testing, viral load testing, hospital admission, emergency services, and post-exposure prophylaxis after a potential exposure to HIV. The infant's blood samples were tested to determine viral load by polymerase chain reaction (PCR) test at six weeks, three months, six months, and 12 months after the birth. The child born to an HIV-positive mother was prophylactically administered ART. The clinical data of the infant, i.e., birth weight, sex, and HIV testing details was recorded on various occasions. 

Data was analyzed using SPSS software, version 22.0 (IBM Corp., Armonk, NY). The data on patient's demographics and clinical results was measured in terms of mean values and frequencies using descriptive statistics. The linear trend of HIV cases in women was analyzed using linear regression analysis. A p-value of less than 0.05 was considered significant.

## Results

In this study, a total of 197 pregnant women with HIV/AIDS presented in a period of 15 years from 2008 to 2022. The mean age of women was 29.5 ± 6.4 years. Most of the women, 114 (57.6%), were between 21 and 30 years. Seven cases (3.4%) were found to be younger than 20 years, while another 13 (6.8%) women were 41 years or above age. When HIV RNA load was assessed in study women, it was witnessed that 67 (34.1%) had undetectable load while 130 (65.8%) had detectable viral load. Similarly, CD4 cell count was less than 200 in 26 (13.4%) cases, between 200 and 499 in 92 (46.4%) cases, while it was > 500 in 79 (40.2%) cases (Table 1).

**Table 1:** Baseline characteristics of mothers and babies (n=197) HIV: Human immunodeficiency virus; CD4: Cluster of differentiation 4.

Characteristics	N	%
Age (years)		
Up to 20	7	3.4%
21 to 30	114	57.6%
31 to 40	63	32.2%
41 or above	13	6.8%
Mean ± SD	29.5 ± 6.4	
HIV RNA load		
Undetectable (<50)	67	34.1%
Detectable	130	65.8%
CD4 cell count (cells/mm^3^)		
<200	26	13.4%
200 to 499	92	46.4%
> 500	79	40.2%
Baby sex (n=127)		
Male	52	40.1%
Female	75	59.9%
Birth weight (kg) n=127		
Mean ± SD	2.8 ± 0.5	

**Figure 1:**
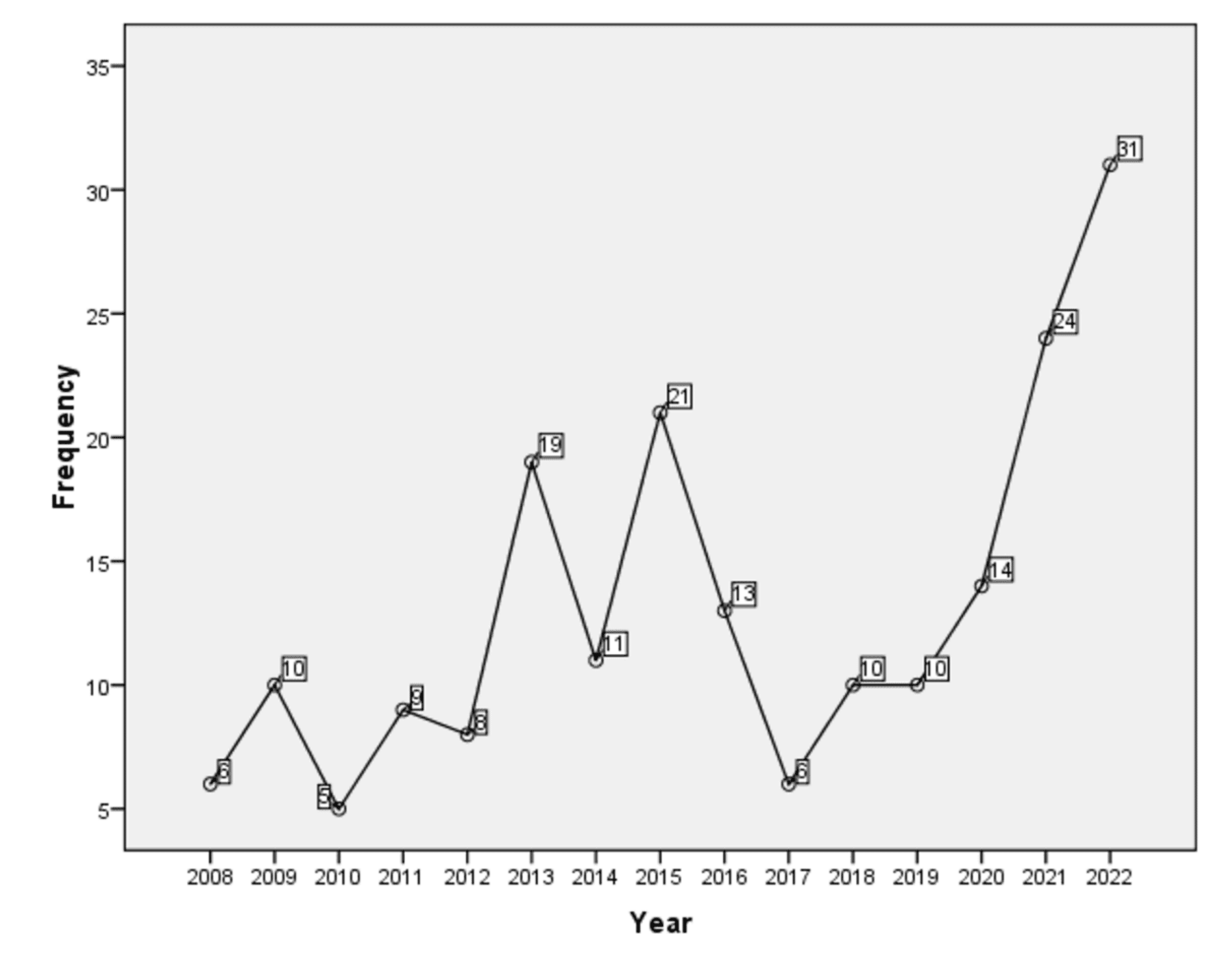
Year wise frequency of HIV/AIDS cases in pregnant women

The frequency of HIV/AIDS cases varied throughout the years. In 2008, there were six (3.0%), and in 2009, there were 10 (5.1%) women who were diagnosed with HIV during pregnancy. In the year 2010, a similar number of cases, five (2.5%), while in the year 2011, there were nine (4.5%) cases of HIV. In 2012, there were 8 (8.0%) cases, which jumped to 19 (9.6%) in the year 2013. In 2014, there were 11 (5.5%) cases of HIV observed, while in 2015, the number rose to 21 (10.6%). In 2016, there were 13 (6.5%) cases who presented with HIV in the MCH center, while in 2017, the number dropped to six (3.0%). The number of women with HIV was 10 (5.0%) cases each in the years 2018 and 2019. In 2020, there were 14 (7.0%) cases of HIV, while in 2021, the number was 24 (12.2%), which jumped to 31 (15.7%) in the year 2022 (Figure 1).

**Figure 2:**
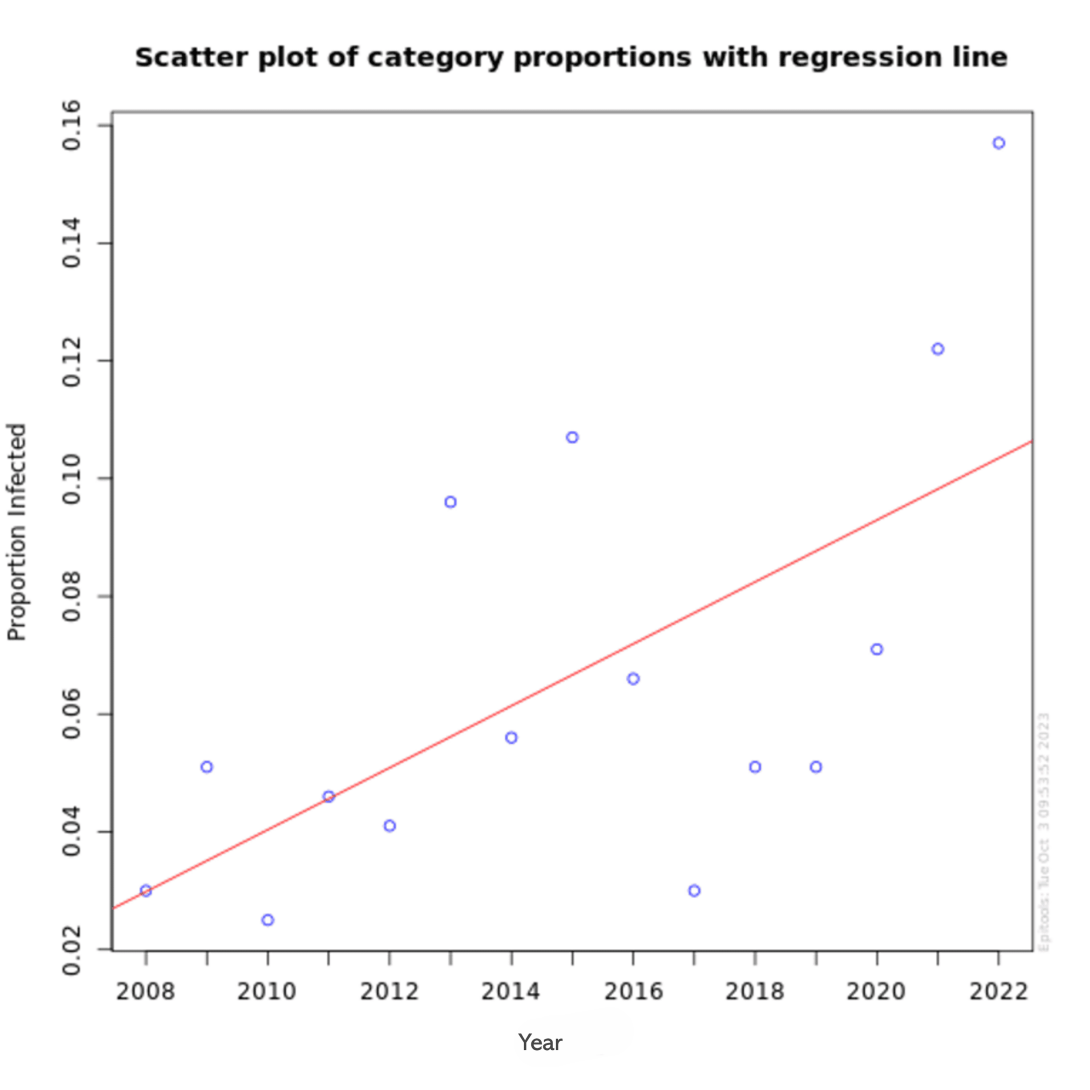
Linear trend of HIV in pregnant women presenting to MCH center, PIMS, Islamabad Trends linear regression value (p-value = 0.014). MCH: Mother and Child Health Care; PIMS: Pakistan Institute of Medical Sciences.

Figure 2 illustrates the linear trend in the number of HIV cases among pregnant women presenting to the MCH Center at PIMS Islamabad over 15 years from 2008 to 2022. A linear regression analysis was performed to evaluate the trend over the years. The analysis showed a statistically significant increase in the number of HIV cases, with a coefficient of 1.04 (standard error = 0.36, p = 0.014).

Out of the total 197 women with HIV, vertical transmission was witnessed in two (1.0%) babies. Most of the babies born alive were negative for HIV transmission 83 (42.1%) in this study. There were 65 (33.1%) women who presented for antenatal care and got screened for HIV, however, they were lost to follow-up and in most cases, the families or women were irresponsive and did not answer the phone calls despite being contacted on many occasions. There were miscarriages observed in 22 (11.1%) study women, while stillbirths were noted in four (2.0%) cases. Of the total, 11 (5.6%) women expelled or aborted their pregnancy, while 10 (5.0%) babies died soon after birth or within a month of delivery (Table 2).

**Table 2:** Outcome of pregnancy (n=197)

Characteristics	N	%
Vertical transmission (positive baby)	2	1.0%
Negative baby	83	42.1%
Lost to follow-up	65	33.1%
Miscarriage	22	11.1%
Aborted/Expelled pregnancy	11	5.6%
Baby expired	10	5.0%
Stillbirths	4	2.0%

## Discussion

This study found a linear trend of increase in HIV/AIDS cases in pregnant women presenting to the Obstetrics and Gynecology Department at the MCH Center, PIMS, Islamabad. The vertical transmission of HIV from mother to newborn was noted in 1.0% of the current study cases. Though the rates of vertical transmission are in line with international data, the overall trend of women presenting with HIV was found to be increasing year by year. All the women when diagnosed or found pregnant with HIV were started on Highly Active Antiretroviral Therapy (HAART) treatment, this may be the reason for the reduced vertical transmission of HIV from mothers to babies. 

Previous evidence suggests vertical transmission in less than 2% of HIV cases from mothers to babies [13]; this is comparable with the current study findings of 1.0% vertical transmission with loss to follow-up among almost half the women. Indeed, the lower rate of vertical transmission observed in our study, at 1.0%, could be influenced by the fact that this is a single-center study, which may not fully represent the national prevalence of vertical transmission of HIV. Single-center studies often reflect localized practices and patient populations, which can result in outcomes that differ from broader, multi-center, or national studies. A study by Myaphi and colleagues [14] from South Africa witnessed vertical transmission of 9.4% in their study of pregnant women with HIV infection. They concluded that supplemental testing for HIV during pregnancy led to filtration of positive cases, and inappropriate screening may undermine the effort to avoid mother-to-child transmission [13,14]. The European Collaborative Study on Mother-to-Child Transmission of HIV Infection in the Era of Highly Active Antiretroviral Therapy reported vertical transmission of 2.87%. They noted that HAART therapy increased from under 10.0% in the 1990s to 92.0% in 2003-2004. The authors concluded that it is appropriate to offer an elective caesarean section delivery to all HIV-infected women, even in areas where HAART is available and especially in patients having a detectable viral load [15]. A pooled analysis from India reported a prevalence of 8.6% of mother-to-child transmission of HIV; however, they reported variation in prevalence from various regions. They witnessed a prevalence of as low as 3.5% in Gujrat, 7.1% in Mumbai, as high as 14.2% in Tamilnadu, and 29.4% in Northern Bengal [16-20]. One South African study witnessed a rate of 245 in-utero paediatric HIV infections per 100,000 live births, which ranged from 263-294 infections per 100,000 live births [21]. The high rates of vertical transmission suggest irresponsible behavior and non-compliance with HAART and other preventive measures during pregnancy and delivery. These mothers need a close liaison with healthcare facilities and frequent face-to-face or digital follow-ups. 

In the present study, the majority of women were between 21 and 30 years old, with an average age of 29.5. Many others have also shared these stats, with Potty et al. also reporting a median age of 30 years, with the majority of their study women between 20 and 30 years old. This is in line with the general age of marriage in women in most parts of the world [22]. 

In the current study, the most concerning finding is the linear increasing trend of women presenting with HIV in pregnancy, which began at 3.0% in 2008 and jumped five times to up to 15.7% in 2022. It was noted that the prevalence of HIV/AIDS was no or very low till the late 90s and early 2000s, however, cases started coming after 2000 and this has now inflated in recent times. Several factors, including the lack of awareness, poor literacy rates, unsafe medical practices, and high-risk behaviors such as unprotected sex and drug use, may influence the increase in HIV cases observed in our study. These factors are consistent with national trends in Pakistan, where a significant rise in HIV cases has been documented [23]. The study by Aizaz et al. highlights similar challenges at the national level, emphasizing the role of unhygienic medical practices, unsafe blood transfusions, and inadequate awareness as critical contributors to the rising incidence of HIV across the country [23].

The HIV program in the country and program managers need to monitor the spread of HIV internally and externally and filter out the etiology of transmission so that the increasing rates can be controlled. One such proven recommendation for women with HIV is not to breastfeed the baby [24].

Another mode of transmission is via breastfeeding; many studies have previously highlighted preventive measures at the personal, healthcare, and community level to avoid this mode of HIV infection [25]. In the present study, no baby contracted HIV via breastfeeding. Irrespective of the mode of transmission, it is a serious threat and a grave public health problem. Investigators and subject specialists recommend HAART treatment during pregnancy for the prevention of vertical transmission of HIV, while to reduce transmission via breastfeeding, there is a need to avoid breastfeeding the baby as well as control the home environment where the child is growing [26].

In Pakistan, where traditional long-term breastfeeding practices are a norm, it is impossible to discourage it. However, HAART in HIV-positive pregnant women is a viable possibility, thus these women should be made 100% compliant with ART therapy [27]. The ART therapy needs to be continued and uplifted during the breastfeeding period as well to reduce the chances of horizontal transmission. The rates of malnutrition and common childhood infections are very prevalent in developing country settings, in an already compromised health atmosphere the newborns contracting HIV have a very bleak prognosis due to severe morbidity and mortality risks [28,29].

The strengths of this study include its 15-year data span, providing a comprehensive analysis of trends in HIV transmission among pregnant women, and its detailed focus on the outcomes of vertical transmission within a single tertiary care setting, which is the first of its type from Pakistan. 

There are some limitations of this study as well. Firstly, the transmission of data was available for early outcomes for a year and a half only, and no long-term data could be measured after that since many patients were lost to follow-up. The second limitation was related to specific trends and vertical transmission, while etiological factors related to HIV in women and their details about treatment and effect on their physical and psychological health were out of the scope of this study. Additionally, we could not explore the psychosocial aspect of HIV among the patients, and it was not known how the families and friends' circle took their status of HIV. Future studies could address these limitations by conducting a prospective, longitudinal study. 

## Conclusions

It is concluded that a linear trend of increase in the cases of HIV in pregnant women was noted, which is an alarming public health emergency. Moreover, the rate of vertical transmission was found to be 1%, proving that maybe HAART therapy and appropriate preventive measures have a role in the reduction of the risk of mother-to-baby transmission of HIV. Further large-scale studies on HIV are needed to measure the etiological and clinical factors of women in pregnancy. Moreover, horizontal and vertical transmission also needs to be taken into account. It is suggested that appropriate health education and counselling regarding safe sex practices are required to be taught to young women and men in our society. Expatriates and frequently traveling men and women should be advised pre-wedding screening for HIV/AIDS. Also, immigration counters may take samples of people arriving in the country after a long period.

## References

[1] Getaneh T, Dessie G, Desta M (2022). Early diagnosis, vertical transmission of HIV and its associated factors among exposed infants after implementation of the Option B+ regime in Ethiopia: a systematic review and meta-analysis. IJID Reg.

[2] (2024). HIV/AIDS in the World. https://www.amfar.org/about-hiv-aids/statistics-worldwide/.

[3] Di Biagio A, Taramasso L, Gustinetti G (2019). Missed opportunities to prevent mother-to-child transmission of HIV in Italy. HIV Med.

[4] Jaggi R, Sharma R, Majora N, Gupta S (2021). Retrospective eleven years study of PPTCT programme in SMGS Hospital, Jammu. Annals of the Romanian Society for Cell Biology.

[5] Chasekwa B, Ntozini R, Church JA (2022). Prevalence, risk factors and short-term consequences of adverse birth outcomes in Zimbabwean pregnant women: a secondary analysis of a cluster-randomized trial. Int J Epidemiol.

[6] Yang X, Su B, Zhang X, Liu Y, Wu H, Zhang T (2020). Incomplete immune reconstitution in HIV/AIDS patients on antiretroviral therapy: challenges of immunological non-responders. J Leukoc Biol.

[7] Almeida FJ, Kochi C, Sáfadi MA (2019). Influence of the antiretroviral therapy on the growth pattern of children and adolescents living with HIV/AIDS. J Pediatr (Rio J).

[8] Florina MI, Alexandra LG, Carmen DO, Florina BA, Isabela LO, Alexandra LA, Carmen MA (2021). Analysis of the newborns from HIV positive mothers in a period of three years from the regional HIV/Aids Center Iasi, Romania. The Medical-Surgical Journal.

[9] Siddiqui AR, Ali Nathwani A, Abidi SH (2020). Investigation of an extensive outbreak of HIV infection among children in Sindh, Pakistan: protocol for a matched case-control study. BMJ Open.

[10] Mir F, Mahmood F, Siddiqui AR (2020). HIV infection predominantly affecting children in Sindh, Pakistan, 2019: a cross-sectional study of an outbreak. The Lancet Infectious Diseases.

[11] Marcu EA, Dinescu SN, Pădureanu V, Dumitrescu F, Diaconu R (2022). Perinatal exposure to HIV infection: the experience of Craiova Regional Centre, Romania. Healthcare (Basel).

[12] (2024). National AIDS Control Programme. https://www.nih.org.pk/public/national-aids-control-programme.

[13] (2024). HIV Surveillance Report: 2002; vol. 14. https://stacks.cdc.gov/view/cdc/149081.

[14] Mayaphi SH, Martin DJ, Quinn TC, Stoltz AC (2019). Vertical transmission of HIV among pregnant women who initially had false-negative rapid HIV tests in four South African antenatal clinics. PLoS One.

[15] (2005). Mother-to-child transmission of HIV infection in the era of highly active antiretroviral therapy. Clin Infect Dis.

[16] Bhatta M, Dutta N, Nandi S, Dutta S, Saha MK (2020). Mother-to-child HIV transmission and its correlates in India: systematic review and meta-analysis. BMC Pregnancy Childbirth.

[17] Gupta A, Gupte N, Sastry J (2007). Mother-to-child transmission of HIV among women who chose not to exclusively breastfeed their infants in Pune, India. Indian J Med Res.

[18] Ahir SP, Chavan V, Kerkar S, Samant-Mavani P, Nanavati R, Mehta PR, Mania-Pramanik J (2013). Antiretroviral treatment, viral load of mothers & perinatal HIV transmission in Mumbai, India. Indian J Med Res.

[19] Chaudhuri S, Mundle M, Konar H, Das C, Talukdar A, Ghosh US (2010). Utilization of therapeutic intervention to prevent mother to child transmission of HIV in a teaching hospital in Kolkata, India. J Obstet Gynaecol Res.

[20] Goga A, Chirinda W, Ngandu NK (2018). Closing the gaps to eliminate mother-to-child transmission of HIV (MTCT) in South Africa: understanding MTCT case rates, factors that hinder the monitoring and attainment of targets, and potential game changers. S Afr Med J.

[21] Pai NP, Barick R, Tulsky JP (2008). Impact of round-the-clock, rapid oral fluid HIV testing of women in labor in rural India. PLoS Med.

[22] Potty RS, Bradley JE, Ramesh BM (2012). Is HIV prevalence declining in southern India? Evidence from two rounds of general population surveys in Bagalkot District, Karnataka. Sex Transm Dis.

[23] Aizaz M, Abbas FA, Abbas A, Tabassum S, Obeagu EI (2023). Alarming rise in HIV cases in Pakistan: challenges and future recommendations at hand. Health Sci Rep.

[24] Marfani WB, Khan HA, Sadiq M, Outani O (2022). The rise in HIV cases in Pakistan: prospective implications and approaches. Ann Med Surg (Lond).

[25] Myburgh D, Rabie H, Slogrove AL, Edson C, Cotton MF, Dramowski A (2020). Horizontal HIV transmission to children of HIV-uninfected mothers: a case series and review of the global literature. Int J Infect Dis.

[26] le Roux SM, Abrams EJ, Nguyen KK, Myer L (2019). HIV incidence during breastfeeding and mother-to-child transmission in Cape Town, South Africa. AIDS.

[27] Pontiki G, Lykeridou K, Vivilaki VG (2022). Good communication and trust relationships with women are critical for HIV positive pregnant woman. Eur J Midwifery.

[28] Müller O, Krawinkel M (2005). Malnutrition and health in developing countries. CMAJ.

[29] Modi S, Chiu A, Ng'eno B, Kellerman SE, Sugandhi N, Muhe L (2013). Understanding the contribution of common childhood illnesses and opportunistic infections to morbidity and mortality in children living with HIV in resource-limited settings. AIDS.

